# The embodied brain: towards a radical embodied cognitive neuroscience

**DOI:** 10.3389/fnhum.2015.00237

**Published:** 2015-05-06

**Authors:** Julian Kiverstein, Mark Miller

**Affiliations:** ^1^Institute of Logic, Language and Computation, University of AmsterdamAmsterdam, Netherlands; ^2^School of Philosophy, Psychology and Language Sciences, University of EdinburghEdinburgh, UK

**Keywords:** emotion cognition interactions, psychological constructionism, salience network, embodied cognition, affordances, dynamical systems theory

## Abstract

In this programmatic paper we explain why a radical embodied cognitive neuroscience is needed. We argue for such a claim based on problems that have arisen in cognitive neuroscience for the project of localizing function to specific brain structures. The problems come from research concerned with functional and structural connectivity that strongly suggests that the function a brain region serves is dynamic, and changes over time. We argue that in order to determine the function of a specific brain area, neuroscientists need to zoom out and look at the larger organism-environment system. We therefore argue that instead of looking to cognitive psychology for an analysis of psychological functions, cognitive neuroscience should look to an ecological dynamical psychology. A second aim of our paper is to develop an account of embodied cognition based on the inseparability of cognitive and emotional processing in the brain. We argue that emotions are best understood in terms of action readiness (Frijda, 1986, 2007) in the context of the organism’s ongoing skillful engagement with the environment (Rietveld, 2008; Bruineberg and Rietveld, 2014; Kiverstein and Rietveld, 2015, forthcoming). States of action readiness involve the whole living body of the organism, and are elicited by possibilities for action in the environment that matter to the organism. Since emotion and cognition are inseparable processes in the brain it follows that what is true of emotion is also true of cognition. Cognitive processes are likewise processes taking place in the whole living body of an organism as it engages with relevant possibilities for action.

## Introduction

Radical embodied cognitive science is a relatively new branch of cognitive science that looks to ecological psychology and dynamical systems theory to understand the contribution of bodily capacities to cognitive processes (Chemero, 2009; Barrett, 2011). It is “radical” in claiming that cognitive scientists need new conceptual tools if they are to understand the ways in which cognition depends on the body in its interaction with the environment. The classical conception of the human mind as working according to the same principles as a digital computer encourages us to think of the body and the environment as providing at best inputs to, and receiving outputs from cognitive processes (Rupert, 2009). Embodied approaches to cognitive science by contrast stress the many and varied ways in which an animal’s environmental niche offers resources for the animal to act on. The individual has bodily skills and abilities that are refined and perfected through practice for dealing adequately with the possibilities for action the environment offers. The explanatory tools cognitive science deploys must do justice to the essential contributions of bodily skills and environmental affordances to cognitive behavior. They must account for the ways in which the individual is able to expertly coordinate their behavior with a dynamically changing environment. Ecological psychology and dynamical systems theory provide the tools to meet this challenge. Moreover the conceptual tools these sciences offer are arguably better suited to explaining the complex, dynamical interactions between an animal and its environment than the standard computational tools of cognitive science. This gives us a pragmatic reason to add these explanatory frameworks to the explanatory toolkit of cognitive science.

Our aim in this paper will be to argue that cognitive neuroscience should look to ecological dynamical psychology for an understanding of the psychological functions the brain performs. The classical approach to cognitive science encourages the following view of the division of labor between psychology and neuroscience. Cognitive psychology provides analyses of the cognitive operations an individual must perform in order to carry out a cognitive task. Cognitive neuroscience then seeks to determine how these cognitive operations are carried out by brain regions and networks of brain regions. Consider vision as an example. The tasks the visual system performs are commonly decomposed and broken down into early visual processing in which an image is formed, and features of the surfaces in the surrounding environment are represented. At an intermediate stage in visual processing features of surfaces are used to construct object representations. Late processes then use object-based information to put objects into categories (Palmer and Kimchi, 1986). Cognitive neuroscientists design their experiments based on this type of functional decomposition of visual processing in the brain. The aim of their experiments is to use the knowledge we have accumulated from previous experiments to determine how these different stages in visual processing are carried out by the brain. There is no room in this view of the division of labor between psychology and neuroscience for the body and the environment to play anything other than a peripheral role in cognitive processes.

A radical embodied cognitive neuroscience will take its analyses of psychological function not from cognitive psychology, but instead from ecological psychology and dynamical systems theory. The advantage of doing so will be an analysis of psychological function that does justice to the contribution of the bodily capacities and the ecological setting to a given cognitive behavior.

The first part of our paper will point to findings that strongly suggest it is time for cognitive neuroscientists to look elsewhere for their analyses of psychological function than to classical cognitive psychology. We begin by reviewing recent research concerned with large-scale patterns of connectivity in the brain. We take this research to present a major challenge to any analysis of the brain into functionally specialized regions that carry out either emotional or cognitive psychological functions. Such a view of the brain as being made up of functionally specialized regions follows naturally from the view of the division of labor between psychology and neuroscience we sketched above. Cognitive psychology has the job of providing analyses of the functions that are computed in emotional and cognitive processing. Cognitive neuroscience looks to determine how these functions are performed by different regions and networks in the brain (see e.g., Posner et al., 1988). The work we will review on functional and structural connectivity in the brains of humans and other animals supports a different perspective on brain processes in which classical emotional and cognitive brain regions are in constant dynamic interaction. We will show how this work on functional connectivity makes trouble for attempts at localizing either emotional or cognitive functions to specific brain structures.

We argue next that psychological function is better understood at the level of the whole brain-body-environment system. The argument we offer for this second thesis is somewhat circuitous, but necessarily so because it enables us to engage with, and distinguish our proposal from other related theories in the literature on emotion and cognition. We begin by comparing our perspective with that of psychological constructionist (or dimensional) theories of emotion which interpret the integration of cognitive and emotional processes in terms of interactions between domain general neural networks (see e.g., Barrett and Satpute, 2013). We suggest (following arguments developed in Pessoa, 2014) that structure-function mappings are not fixed and static properties of networks. Instead structure-function relationships are dynamic, with the functions a given network performs varying over time in a highly flexible and context-dependent manner. We then argue that the functional contribution of a network is determined by the whole organism in its interaction with an environment that is rich with possibilities for actions. To determine the precise functional contribution of a network to an animal’s behavior we must look at how this network functions in the context of a wider organism-environment system.

The radical rejection of the classical computationalist explanatory framework by advocates of an embodied approach to cognitive science has been taken to call into question the place of the concept of representation in psychological explanation. Hutto and Myin have argued for instance that the dynamic engagement of an animal with the environment doesn’t require the extraction, processing and manipulation of states with semantic or representational content (Hutto and Myin, 2013). We do not engage directly with this issue, but instead focus our arguments on defending the claim that embodied cognition is best studied at the level of the whole brain-body-environment system. We leave the implications of our arguments for the role of representation in psychological explanation as a matter to be discussed on another occasion.

The positive view we develop in this paper will be built up in two stages. First, we argue that cognition and emotion are inseparable processes in the brain. Second, we argue emotion is a dynamic process involving the whole body of the organism. The first two steps in our argument establish the inseparability of emotion and cognition in the brain and the deep dependence of emotional processes on the whole body of the living organism in its practical skilled engagement with the environment. We take these two steps to imply a third step: the conclusion that cognitive processes depend on the whole living body of a person or animal in its practical and skilled engagement with an environment of affordances. Since emotion deeply depends on the living body, so also does cognition.

## Localizing Emotion and Cognition in the Brain

When we ordinarily think of emotion we often think of short-lived, transient episodes that wash over, and sometimes overwhelm us, gradually fading away after a relatively short period of time. Folk psychology makes distinctions between episodes of anger, disgust, fear, happiness, sadness and so on. When we think of our own emotions and those of others we do so using these folk psychological categories. How do these folk psychological emotion categories map onto processes in the brain?

Affective neuroscientists have posited a set of biologically basic emotions such as rage, fear, lust, care, and grief that can be localized to specific and dedicated networks in the human brain (Panksepp, 1998, 2012). Typically these basic emotions are associated with the brain stem, the diencephalon (thalamus and hypothalamus), and limbic structures which are taken to be evolutionarily old, primitive parts of the brain, highly structured at birth and relatively isolated from learning. These parts of the brain are directly connected, and tightly coupled to autonomic, endocrine and immune systems in the body that work together to keep an organism’s body in a state of homeostatic and metabolic equilibrium. They are also taken to be “automatic” in their processing (as contrasted with cognitively controlled processes), and are thought to be critically involved in impulsive behavioral responses such as fear and rage.

Cognitive processes (such as learning and memory, reasoning and planning) are often associated with the phylogenetically newer and more highly evolved cerebral cortex. The neocortex in primates has been described as the “crowning achievement of evolution and the biological substrate of the human mental prowess.” (Rakic, 2009, quoted by Barton, 2012, p. 2098). The primate neocortex for instance shows a fivefold difference in volume when compared to that found in insectivores (Barton and Harvey, 2000). This growth is thought by many to be accompanied by an evolution of higher-cognitive functions. Systems in the prefrontal cortex (PFC) for instance make use of information that has been processed by other parts of the brain to ensure that we, in contrast to our animal ancestors, keep our emotional impulses in check. As the Victorian neurologist John Hughlings-Jackson put it: “the higher nervous arrangements evolved out of the lower keep down those lower, just as a government evolved out of a nation controls as well as directs that nation.” (Jackson, 1884, p. 662, quoted by Parvizi, 2009, p. 354) The frontal lobes exert control over, and suppress our more animal desires, thereby ensuring that we act in ways that are contextually appropriate. These functional processes allow higher mammals to compare possible plans and strategies offline, and make a cost-benefit calculation as to which possible course of action is likely to be the most beneficial in the long run.

This understanding of cognition and emotion leads to a view of the mammalian brain as divided into cognitive “higher” regions (neocortex) and emotional “lower” subcortical regions. This division is perhaps best exemplified in Paul MacLean’s discredited triune model of the mammalian brain (MacLean, 1952, 1990; for criticisms see Swanson, 1983; LeDoux, 2012). The lower, animal parts of the brain are understood (in line with the Hughlings-Jackson “Victorian” narrative) as standing in a linear, and hierarchical relationship to the higher neocortical regions. Why assume however that the only parts of the human brain to undergo change over the course of evolution were those located in the cortex? An alternative co-evolutionary hypothesis is that both cortex and sub-cortex underwent changes in a coordinated fashion. Those brain structures with major anatomical and functional links most likely evolved together (Barton and Harvey, 2000). Barton (2012) discusses for instance how the cerebellum (an area known to be involved in the learning of motor skills) is larger in primates compared with other mammals. He adduces evidence for the threefold co-evolution of the diencephalon, cerebellum and the neocortex. There is also evidence that subregions of the amygdala are substantially more developed in monkeys as compared with rats (Chareyon et al., 2011). Pessoa has suggested, in line with the co-evolutionary hypothesis we just sketched, that this increase in size in the amygdala is likely to be linked to the degree of connectivity with other brain structures (Pessoa, 2014, pp. 413–414).

The old hierarchical model of the higher primate brain and the lower reptilian brain assumes a unidirectional flow of information from lower to higher brain systems. While the higher-brain systems depend on lower-brain systems for their functioning the reverse is not true. Parvizi (2009) offers an important critique of this “corticocentric myopia”. In his words “higher functions of the brain are made possible by a reciprocal interconnection between cortical and subcortical structures rather than being localized only in the upper tip of the vertical neuroaxis” (p. 354). Parvizi describes the connectivity between the cortex and the rest of the brain in terms of “reciprocal interconnection”. This means that there are complex relations of negative and positive feedback that characterize the communication between sub-cortex and cortex. Parvizi suggests that higher brain functions (such as executive control functions) happen in “the loops operating between the cortical areas and “lower” subcortical structures such as the basal ganglia; the basal forebrain; the thalamus; the cerebellum; and the brainstem dopaminergic and noradrenergic systems.” (p. 358, supporting references omitted).

Consider as an example the hypothalamus, a region located just below the thalamus and above the brain stem. The hypothalamus is commonly associated with the coordination of homeostatic mechanisms such as hormonal and behavioral circadian rhythms and neuroendocrine processes. However, it is also bidirectionally connected to the cerebral cortex by at least four pathways that run via the basal forebrain and amygdala; the brainstem and the thalamus (Risold et al., 1997). Barbas and Rempel-Clower (1997) showed that in primates the hypothalamus projects to all regions (orbital, medial and lateral) of the PFC. The hypothalamus functions as a so-called “connectivity hub” that is optimally placed because of its extensive connections to have a near global effect on brain function (Pessoa, 2013, pp. 230–231). Importantly, areas of the PFC (orbital and medial PFC), insular cortex, hippocampus, and amygdala also link back to the hypothalamus. The connections between the hypothalamus and PFC are bidirectional and reciprocal, allowing for rapid coordination and synchronization of activity between “higher” and “lower” brain systems. This coordination allows for cognitive and affective processes to be mobilized together allowing the animal to behave flexibly, and in ways that are adapted to the particularities of a context of activity.

This picture of higher cognitive systems and lower emotional systems as being “vertically” integrated and tightly coordinated strongly argues against a corticocentric myopia. It suggests instead a view of cognitive and emotional processes as strongly interdependent (Lewis, 2005; Stapleton, 2012; Pessoa, 2013; Colombetti, 2014, ch.4). By “interdependent” we mean to refer to the degree to which cortical and sub-cortical systems influence each other. This degree of influence is measured by the information-processing operations the components that make up these systems each perform. The evidence (some of which we have reviewed above) points to a tight coupling or mutual influence between these systems. The operations carried out by components located in the cortex are constantly effecting, and being effected by, the operations that are taking place in components found in the sub-cortical systems. Functional connectivity isn’t just about channeling information between functionally specialized brain regions. Instead it “generates complex system-wide dynamics that enable local regions to participate across a broad range of cognitive and behavioral tasks” (Byrge et al., 2014, p. 395).

Marc Lewis provides a clear example of the dynamical interaction between emotion and cognition in the brain (Lewis, 2005). He discusses the cognitive and neural processes that are engaged when a person experiences “road rage”. We quote him at length because his example makes it clear why it is important to understand this interaction between emotional and cognitive processes as taking place at the level of the whole embodied person in the environment, a point we return to below.

“Mr. Smart slams on the brakes when noticing the proximity of the car in front. Anger arises initially from frustration, as Mr. Smart wants to keep driving fast, but also from a sense of violated entitlement: he is in the left lane and should not have to slow down. Fear may also be triggered by the close call, eliciting further anger because of an intermediate evaluation of unmanly helplessness. These emotions arise rapidly, but they are paralleled by a co-emerging sense of the other driver as intentionally obstructive (and therefore blameworthy). Mr. Smart’s highly focused visual attention, a derivative of anger, takes in the red color of the car ahead, as well as the expensive-looking design, and his anger is amplified by his sense of the unfairness of this show-off blocking his path (based on an implicit memory of some long-forgotten or fantasized rival). A stabilizing angry-anxious state, coupled with ruminative plans for vengeance (perhaps a blast of the horn), anchors attention to the head of the man in front. This lasts for a minute or two while Mr. Smart fashions and modifies plans to pass on the right. However, when the man peers over his shoulder, Mr. Smart evaluates this act as a taunt, generating shame and anger in an elaborated appraisal of humiliation, and calling for extreme action to save his self-image from further subjugation” (Lewis, 2005, p. 175).

For Lewis a change in emotional state is triggered whenever orderly behavior “is interrupted by a perturbation, resulting in a rapid loss of orderliness and an increase in sensitivity to the environment” (Lewis, 2005, p. 174). In Smart’s case the suddenly obstructed lane jolts his body from a state of low arousal (feeling of calmness and flow) to being highly aroused (feelings of fear and anger). Lewis writes: “living systems are like taut springs, ready to respond to small perturbations that are biologically meaningful” (Lewis, 2005, p. 176; see also Kauffman, 1993). Any cognitive or emotional event can be a trigger, so long as it sufficiently perturbs the system into a state of disorder. Once order has been disrupted, sub-cortical and cortical neural systems enter into a series of recursive feed-back loops, activity in the one system amplifying activity in the other (Lewis, 2005, p. 176). Consider for example the positive feed-back loop established between the psychological processes of bodily arousal on the one hand (realized in sub-cortical, limbic systems), and attention and recall (involving regions in the neocortex) on the other. Smart’s feelings of violation at being halted in the fast lane triggers feelings of anger. The arousal generated by the anger motivates and focuses Smart’s attentional resources onto the dangerous and transgressive elements in the environment: the driver of the red sports car. This increased attentional focus highlights the expensiveness of the automobile, which in turn triggers feelings of unfairness, which increase Smart’s feelings of anger. Simultaneously Smart’s angry arousal anchors his recall processes to similar past situations, and memories of past transgressors, which amplifies Smart’s feeling of anger. Smart’s bodily arousal motivates and directs his attentional and recall processes. These cognitive processes in turn intensify and prolong his state of anger arousal.

When the somatic and cognitive processes become appropriately coupled (anger in the form of bodily arousal directing attention and recall, attention and recall sustaining/amplifying bodily arousal) the brain systems supporting these psychological processes begin to settle into stable patterns of activity (Lewis, 2005, p. 177). Just as a group of birds quickly settles into an enduring flocking pattern, so also do Smart’s emotional and cognitive processes temporarily stabilize and settle into a coherent and large scale anger-anxiety state. The “lower” neural processes that track bodily arousal, and the “higher” neural processes associated with attention and memory sustain each other, and generate an enduring emotional-cognitive state.

Lewis’s example is framed in psychological terms, but the reciprocal and mutual influence he describes strongly speaks against any separation of emotion and cognition in the brain. Subcortical and cortical networks non-linearly interact in his example in such a way as to sustain temporary, large-scale patterns of organization over time. One might object that the activity in these networks take shape on the basis of interaction, or communication between functionally specialized brain regions. We think this is exactly the picture of the organization in the brain that is called into question once one rejects corticocentric myopia. In the Smart example, we see how negative and positive feedback loops shift the brain from a state of relative disorder to a temporary, more or less short-lived pattern of global coherence. This shift from disorder to global coherence involves the formation of large scale networks in the brain. The relations of feedback that are critical for the formation of such networks mean that each element is directly or indirectly affecting every other element that makes up the network. This fundamentally challenges a picture in which each element performs a psychological operation (either emotional or cognitive) apart from its interactions with other elements. We suggest instead conceptualizing psychological function at the level of the processes taking place in the large-scale network as a whole. Large-scale networks implement psychological processes that are simultaneously both emotional and cognition in nature. They are in Lewis’s words “amalgams” of emotion and cognition.

## Are Emotional and Cognitive Processes “Psychological Constructs”?

We have now given the argument for the first claim we wish to make in this paper that the brains of animals resist functional decomposition into separable emotional and cognitive components. In this section we begin making the case for the second step in our argument, which is a defense of the claim that the amalgam of emotional and cognitive processes we find in the brain deeply depend upon the whole living body of an organism. We begin by discussing psychological constructionism because research in this tradition would seem, at least at first glance, to lend further support for the view we have been developing. Psychological constructionism grew out of the dimensional theory of emotion. The dimensional theory claims that emotional episodes such as fear, anger, and sadness are combinations of more fundamental dimensions such as arousal (the strength and intensity of an emotion) and valence (the degree of pleasantness or unpleasantness) working in combination with cognitive processes of appraisal The dimensional theory can be traced back to Wundt and has received more recent defense in the work of James Russell and Lisa Feldman-Barrett (Wundt, 1897; Russell, 1980, 2003; Barrett, 2006). More recently, psychological constructionists have begun to argue that emotions do not map onto distinct regions and networks in the brain but are instead the result of dynamic interactions between large-scale networks that compute domain-general functions (Barrett and Satpute, 2013). This looks to be very much in keeping with what was argued in the previous section.

The constructionist theory is often contrasted with categorical or discrete theories of emotion (briefly discussed at the beginning of section Introduction). Discrete theories of emotion posit basic emotions (e.g., anger, fear, sadness, happiness, disgust and surprise) that are species-universal, hardwired, and have unique physiological and neural signatures or profiles (see e.g., Panksepp, 1998; Ekman, 1999; Izard, 2007, 2011). Discrete theories of emotion have unfortunately often subscribed to the problematic division of the brain into emotional and cognitive systems criticized in the previous section (see e.g., Panksepp, 1998). Constructionists add something important to our earlier argument by calling into question the claim that emotions can be mapped onto specific functionally-specialized regions and networks in the brain. They argue instead that the brain is organized into domain-general, distributed functional networks, and emotions are the result of interactions between these networks.

A growing literature supports a view of function-structure mappings in the brain as many-to-many, thereby bolstering the case for a constructionist theory of emotion. There can be no one-to-one mapping of psychological function to anatomical regions or structures because brain regions and structures exhibit extensive pluripotency and degeneracy. Pluripotency refers to the well-established finding that one and the same region (e.g., Broca’s area) can be involved in the performance of multiple functions e.g., language processing, movement preparation, imitation and imagery related tasks (see Anderson, 2010, 2014 for discussion of this and many other examples of pluripotency). Degeneracy refers to the finding that different neural structures can perform one and the same function (Edelman and Gally, 2001; Friston and Price, 2003; Figdor, 2010). Taken together these findings suggest a *many-to-many* mapping of structure to function at the level of brain regions.

This seems to present a challenge to the discrete theory of emotion. Pluripotency and degeneracy strongly suggest that each basic emotion is unlikely to have its own physiological and neural profile. Consider what we now know about the amygdala in this regard, an area that is often referred to as supporting a discrete theory of fear because it has repeatedly been shown to be involved in threat responses in rats and humans (see e.g., LeDoux, 1996; Öhman and Mineka, 2001; for critical discussion see Sander et al., 2003). The amygdala has however also been shown to be active when people are presented with novel, but emotionally neutral stimuli (Moriguchi et al., 2011; Balderston et al., 2011; Blackford et al., 2011). Herry and colleagues for example found increased activity in the amygdala when subjects were presented with unpredictable sequences of tones as compared with predictable sequences (Herry et al., 2007). The amygdala is involved in a wide variety of different functions, including “cognitive” functions such as value representation and decision-making (Sergerie et al., 2008; Pessoa, 2013, ch.2).

Recent meta-analyses have shown that other brain regions associated with emotion such as the anterior insula, pregenual and subgenual anterior cingulate and orbitofrontal cortex also show increases in activity for a variety of different emotion states. Lindquist and colleagues compared the sets of brain regions that were consistently activated in studies of anger, disgust, fear, happiness and sadness. They found six distributed networks that consistently showed up in the studies they analyzed from 1990–2007. The networks and the regions of which they were composed were not associated with particular emotion categories, but were instead found to be active in all studies of emotion experience they analyzed. They found no brain regions that were functionally specialized—every region that was activated for one emotion category was also activated for at least one other emotion category (see Figure 5 in Lindquist et al., 2012 for a useful visual summary of the findings of their meta-analyses). The same brain regions can carry out a variety of distinct psychological operations and belong to different overlapping networks over time (also see Anderson, 2010, 2014; Colombo, 2013). What a brain region does at any given time will depend on the network with which it is affiliated.

Lindquist and colleagues argue their meta-analysis supports a constructionist or dimensional theory of emotions, and challenges discrete theories (which they label “natural kind” theories). Emotion in general has as one of its components bodily arousal which can (but need not) be combined with pleasurable or unpleasant feelings. We will henceforth refer to the latter dimension of emotion as “valence”. Constructionists give this combination of arousal and valence the label “core affect”. Core affect plays a role in homeostasis tracking endocrinal, visceral and muscular changes internal to the body that inform the organism that there is something in the environment of potential relevance or value. Barrett and Satpute (2013) take core affect to be neurally realized by a large-scale intrinsic network that has come to be called the “salience” or “ventral attention” network (Menon and Uddin, 2010). An intrinsic network is a network of widely distributed brain regions whose activations are tightly correlated across time when subjects are at rest, and their attention is not engaged by any external task or stimulus (Seeley et al., 2007; Bressler and Menon, 2010; Raichle, 2010; Yeo et al., 2011). Barrett and Satpute suggest that the salience network is made up of dorsal and ventral subnetworks. The dorsal subnetwork uses homeostatic and metabolic information from the body to guide attention and motor behavior. The ventral subnetwork realizes affective feelings that are experienced by a subject as pleasurable or unpleasant with different degrees of arousal. The salience network carries out “domain-general” functions, which is to say that this network of brain regions is active in a wide range of tasks (i.e., it is not domain or function-specific). What these tasks all share in common is they all require the orienting of attention to homeostatic or metabolically relevant information.

Core affect doesn’t provide the basis for making folk psychological distinctions between emotions. It is a feature common to all of the emotions we ordinarily distinguish between in folk psychology. Core affect takes on the character of different emotions only through the interaction of the salience network with other domain general networks in which such functions as categorization, language processing and executive control takes place. We focus on the role of categorization here since it will prove important for the argument we make for an embodied view of psychological function in the next section of the paper. It also provides an opportunity for us to briefly compare our theory with related work on the embodiment of emotion.

Constructionists argue that it is on the basis of the categorization of core affect that states of bodily arousal are made meaningful and related to a determinate object. Using the meta-analytic studies mentioned above as evidence they have recently argued that categorization takes place in the episodic memory, or default mode network (DMN) of brain regions that reconstructs past experiences for use in current processing (Bar, 2007; Wilson-Mendenhall et al., 2011; Lindquist and Barrett, 2012; Lindquist et al., 2012). Areas of the default-mode network (medial PFC, posterior cingulate cortex/precuneus, medial temporal lobe) were found to be consistently active during a range of emotional states (Kober et al., 2008; Lindquist et al., 2012). Constructionists have proposed the hypothesis that the DMN may function to model probabilistically the causes of current core affective changes (Lindquist et al., 2012, p. 125). The result of such models of the causes of core affect is the categorization of core affect as an instance of anger, fear, sadness or whatever (see also Barrett and Bar, 2009).

As already noted, this hypothesis certainly makes a good fit with the findings we reviewed in the previous section of extensive connectivity between emotional and cognitive brain regions. Emotion is the outcome of interaction between multiple psychological components, each associated with assemblies of neurons within distributed networks. Lindquist and colleagues write that “these networks combine and constrain one another like ingredients in a recipe, influencing and shaping one another in real time according to the principles of constraint satisfaction” (p.126). However, we shall argue that even this picture of function-to-structure mapping may need to be revised in the light of the arguments we made above in section one.

Constructionists subscribe to a mechanistic view of psychological functions whereby emotion is decomposable into basic psychological operations which can then be mapped onto distinguishable networks or “flexible assemblies of neurons” that “fire together in a probabilistic way” (Lindquist and Barrett, 2012). We suggest however that functional connectivity may make trouble for such a functional decomposition, just as it did for attempts at localizing emotional or cognitive functions to specific components. We saw in the Smart example how large-scale networks form in the brain through positive and negative feedback. This means that either directly through local anatomical connections, or indirectly through long-range connections, every brain region has the potential to influence every other brain region within a given network. The function and operations a particular region carries out will be determined by (but also determine) its interactions with the other elements to which it is connected in a network.

In this section, we’ve seen how the same region can play a role in carrying out very different functions over time. In order to determine what function a given region is performing we need to look at the network with which it is affiliated, but this is something that varies over time. Pessoa has suggested that “In the extreme, two networks may involve the exact same regions interacting with each other in distinct ways across time” (Pessoa, 2014, p. 408). The function of a region is thus not a fixed and static property, but is dynamic and context-dependent, varying with the network in which the region is functioning. A version of the problems we have raised with localizing function to structure may therefore also arise at the organizational level of networks. The finding that there is no one-to-one mapping of structure to function might also generalize to the domain-general networks appealed to by psychological constructionists to explain emotion in the brain. The function of a given intrinsic network may also be dynamic, with very different networks making the same functional contribution to behavior at different times (Pessoa, 2014; Fazelpour and Thompson, 2015). The same network of brain regions may contribute differently to behavior because of the way in which the elements of which it is composed are interacting.

The structure-function relation for networks, and not only for brain regions is thus also in dynamic flux. Pessoa has suggested that in order to determine the precise functional contribution of a given network we may need to look at “global variables” such as neurotransmitters, bodily arousal, and slow-wave potentials (2014, p. 408). The function a given network performs is dependent upon the wider context in which the network is active. How should we think about this context-dependence? We shall argue the context we need to take into account may include the rest of the body in its interactions with the environment, as is argued in radical embodied cognitive science. The mechanistic style of explanation that constructionists employ assumes however that networks have fixed domain-general functions. We saw this for instance in the constructionist proposal to divide the salience network into ventral and dorsal subnetworks with ventral regions directing the selection of visceromotor responses, and the dorsal parts being assigned the function of spatial orientation and motor selection. The research we have reviewed on functional connectivity challenges any such view of structure-functions mappings as fixed and permanent.

Is this simply a complication in the mechanistic theory of emotion that constructionists propose, or does it constitute a more serious challenge? In the next section we suggest it may be interpreted as supporting an embodied interpretation of the results reviewed above. Once we start to admit the role of global variables such as activity in neurotransmitter systems and valenced states of bodily arousal in influencing the functioning of a given network, why think of the boundary between the brain and the rest of the body as a sort of “magical membrane” (Hurley, 2010)? Why think that the factors that influence the function of a network reside only inside of the brain? In the next section we argue that bodily states of arousal and valence (which manifest as changes in the body’s vascular, visceral and motor systems) shift the brain from state of relative disorder to temporary patterns of large scale coherence. The environment elicits patterns of action readiness that manifest in the body in the form of states of arousal and valence. We take these two points to establish the main claim of our paper that to understand the psychological function of a large scale network requires neuroscientists to pay attention to the whole organism-environment system.

## The Deep Dependence of Emotion and Cognition on the Living Body

The constructionist, dimensional theory of emotion we discussed in the previous section conceives of emotional experience as the product of the interaction between different components. We’ve discussed the core affect and situated conceptualization networks above. Bodily arousal and valence (core affect) both occur as part of the life-regulation, homeostatic and metabolic processes of an organism, a being that strives to resist disorder and disintegration in its interactions with the environment (Spinoza, 1677/1894, *Ethics* III, 6 and 7; Thompson, 2007, part 2; Colombetti, 2014). We follow Giovanna Colombetti in arguing that these states of the living body provide an organism with a means of evaluating and appraising aspects of its surrounding environment in terms of their relevance or significance for the organism (Colombetti, 2014). “Relevance” is determined by the organism in relation to what the phenomenological philosopher Merleau-Ponty described as the “organism’s proper manner of realizing equilibrium” with the environment (Merleau-Ponty, 1942/1963, p. 154, also see Bruineberg and Rietveld, 2014). When Merleau-Ponty writes of the organism “realizing” equilibrium with the environment, we take him to be referring to situations in which the organism is coping “smoothly” with the environment (to borrow a phrase from Dreyfus, 1991). Crucially, the organism never fully accomplishes equilibrium with the environment so long as it is alive. There is always room for further improvement. As long as the organism has needs and desires it will always be in a state of a state of relative disequilibrium with the environment, a metastable state (Kelso, 2012; Bruineberg and Rietveld, 2014). Living systems act so as to reduce this disequilibrium thereby improving their situation, and taking them closer towards a state of equilibrium with the environment.

One of the core components of bodily affect is what the psychologist Nico Frijda called “action readiness” (Frijda, 1986, 2007). Affect makes the organism ready to act in ways that improve its grip on the situation in which it finds itself (Rietveld, 2008; Bruineberg and Rietveld, 2014; Kiverstein and Rietveld, 2015, forthcoming). The states of bodily arousal that are either negatively or positively valenced we take to be patterns of bodily action readiness. Constructionists also take core affect to be the “body’s way of representing whether objects in the environment are valuable or not in a given context” (Lindquist et al., 2012, p. 124). The core affect network is described as orienting the “brain’s processing capacity towards the most homeostatically-relevant and metabolically-relevant information—it performs a body-based source of attention within the human brain” (Barrett and Satpute, 2013, p. 366). This is all very much in keeping with the view we have just outlined, but with a number of crucial differences. We argue that this orientation to what is relevant in the environment should be conceived of as action readiness, where the latter needs to be understood in the context of a whole animal-environment system.

The living organism always finds itself in an environment offering many possibilities for actions. From these possibilities some are singled out as important to the organism because they are possibilities that elicit an action-readiness in the organism. The organism is drawn to act on those affordances that bring the organism closer to equilibrium with the environment, and move the organism further away from a state of disequilibrium. We are suggesting that interoceptive areas of the brain track changes in patterns of action readiness in the body of the organism as a whole. These bodily changes reflect the organism’s state of relative equilibrium with the environment. When the body of the organism is aroused by some opportunity or challenge in the environment, the effect on the large-scale patterns of activity in the brain is that of destabilizing and disrupting the self-sustaining pattern of organization that has temporarily taken form. In the terminology of dynamical systems theory, the brain is caused to shift out of one attractor state. The brain then settles into new large scale patterns of activity (a new attractor state) that makes the organism ready to act in ways that reduce its disequilibrium with the environment.

In our view, bodily arousal in the form of action readiness already includes some appraisal or evaluation of the environment. The body of the organism is aroused in particular way by opportunities or challenges the environment offers that matter to the organism. Due to the organism’s skills and abilities that have been trained up in the past, the organism is already prepared to do what needs to be done to improve its situation in the world. Constructionists by contrast conceive of core affect not as states of action readiness, but more along the lines of raw sensations that are only given meaning through the cognitive process of situated conceptualization. Lindquist and colleagues describe categorization as functioning like a “chisel, leading people to attend to certain features in a sensory array and to ignore certain others.” Categorization is said to take place in a network of brain regions (the DMN) that are engaged when remembering personal experiences (episodic memory) and when imagining future events (prospection) (Bar, 2007; Buckner et al., 2008). Categorization is hypothesized to take the form of representation of prior experiences (Barsalou, 2003). These prior experiences are used to infer what the most likely cause of the current affective changes in one’s body might be, and it is this inference that allows for the integration of internal changes in bodily experience, and external sensory perception into a “meaningful psychological moment” (Lindquist et al., 2012, p. 124).

Constructionists might seem to be making common cause with recent embodied theorists of emotion such as Paula Niedenthal who take perceiving and thinking about emotion to involve the perceptual, somatovisceral and motoric reenactment or embodiment of the emotion in oneself. Like constructionists, Niedenthal also takes the concepts involved in thinking about emotion to be modal concepts involving a re-experiencing of past experiences. Consider for instance smiling. There is a clear difference between a felt smile and a false smile, a difference it turns out that can be traced to ways in which the muscles around the eye (the *orbicularis* oculi) contract (Ekman and Friesen, 1982). This so-called Duchenne marker is found in felt, but not in false smiles, and is apparently precisely localizable in the brain (Ekman et al., 1990, though one might question this in the light of the arguments given above). Niedenthal and colleagues have argued that the processes involved in recognizing a false from a felt smile in other people might likewise involve one unconsciously simulating offline the very same muscle movements one makes when genuinely or non-genuinely smiling (Niedenthal et al., 2010). Emotion is thus embodied because experiencing emotion, thinking about emotion, and recognizing emotion in others all require one to literally embody the emotion oneself in one’s posture, expression, movements and gestures.

This is an intriguing idea, and the evidence for it is strong. However, there are a number of important differences between Niedenthal’s embodied theory of emotion, constructionism, and our embodied theory. For Niedenthal, folk psychological distinctions between emotions can be precisely mapped onto physiological states which can in turn be correlated with populations of neurons found in sensory, motor and affective regions of the brain (Niedenthal, 2007). She is careful to add that the re-experiencing of physiological states involved in perceiving and thinking about emotion (be it one’s own or other peoples) need not be real physiological states of the body. Simulations of these physiological changes will do. The changes taking place in the living body of the organism turn out to be merely peripheral, and all the real action required for generating an embodied emotion takes place in the brain. We argue by contrast that states of the whole living body in the form of action-readiness drive the meta-stable, large-scale pattern of activations that take shape in the brain. Emotion is embodied because it is realized in states of action readiness that mobilize the organism, orienting the body to relevant possibilities and challenges.

Constructionists would, we suspect, also disagree with Niedenthal’s embodied theory. She seems to be committed to a discrete theory of emotion according to which distinct physiological and neural states are associated with different basic emotions. Russell (2003) and Barrett (2006) have both argued against a mapping of emotions one-to-one onto behavioral expressions of the kind appealed to in Niedenthal’s research. They argue for instance that behavioral expressions of emotion are enormously context-sensitive and exhibit massive situational variance. For example, in some situations I may express my sadness by crying, while in many other situations I may find this sort of open expression of feeling to be inappropriate. We agree with Colombetti (2014), ch.2 however that a dynamical account of emotions of the type we have been proposing, can account for this context-sensitivity without completely rejecting a discrete theory of emotion. Colombetti argues (and we agree) that emotional episodes may be mapped onto “relatively stable patterns of brain and bodily (including behavioral and expressive) processes.” (Colombetti, 2014, p. 48).

The role of past experience in orienting an organism to what is relevant looks very different when viewed from the perspective of the whole living animal in interaction with its environment. Constructionists describe the psychological process of situated conceptualization as involving the reenactment of past experiences, which leads to an understanding of the cause of one’s current bodily state of core affect, and creates “a meaningful mental moment in the present” (Barrett and Satpute, 2013, p. 367). Emotion is responsible for giving an organism a meaningful experience of the environment just as we argue, but it does so only with the mediation of processes of situated conceptualization. We’ve suggested by contrast that affect in the form of action readiness orients the organism to the possibilities for action that matter most to the organism at the time. The organism finds itself ready to deal adequately with the affordances of the environment, but it does so in large part because of its past experience.

Particularly important are the skills and abilities built up over long period of repeatedly encountering and dealing with the same or similar situations. In sports and music for instance “training, repetition and drill is the concrete foundations on which the structure of play gets erected (Noë, 2009, p. 118). However it is not only in these socio-cultural domains that practice matters; an animal’s adequate dealings with the affordances of the environment is always a matter of skill. An animal can respond to an affordance well or badly, and it can get better over time at doing so (Rietveld, 2008). This past history of recurrent interactions with the environment is necessary we suggest for correctly anticipating the outcomes of one’s current interactions with the environment. Past experience thus explains how the animal is currently ready to respond adequately to relevant possibilities for action. It is on the basis of this action readiness (which we have just argued enfolds the organism’s past experiences) that the organism gives meaning to the environment, and certain possibilities for action stand out as immediately relevant to the organism now. We therefore agree with constructionists that past experience plays an important role in creating a meaningful moment of emotional experience. We disagree however about the form this meaningful moment of experience takes. We argue it takes the form of the whole organism being ready to deal adequately with the relevant affordances of its environment.

This in turn suggests a different interpretation of the constructionist finding that the grouping of areas that makes up the DMN are consistently active for a range of different emotional experiences. We speculate that the spontaneous activity found in this population of neurons gives the organism the ability to accurately and precisely *anticipate* the outcome of its interactions with the environment. (For more details on how we would understand these anticipatory processes we direct you to Bruineberg and Rietveld (2014)).

## Conclusion

Our goals in this paper have been twofold. First we wished to show that cognitive neuroscience may need a different account of cognitive function to that which cognitive psychology supplies. Second we wanted to show that ecological psychology and dynamical systems theory under the heading of radical embodied cognitive science may be able to provide such an account of cognitive function. However if we do look to embodied cognitive science to play this role, this means giving up on a brain-centerd view of cognitive function. We will no longer be able to claim that the brain is the organ of the mind. Instead we will need to think about mind and the cognitive processes that make up the mind at the level of the whole brain-body-environment system. Let us briefly recap our argument.

We began by reviewing some of the problems cognitive neuroscientists have run into in mapping emotional and cognitive functions onto discrete and separate structures in the brain. Instead of discrete brain regions and networks performing specialized emotional or cognitive computational operations, we have discussed evidence that points to extensive mutual influence between classical emotional and cognitive areas of the brain. We then argued that this makes trouble for any attempt to localize function in specific brain areas. We turned our attention next to dimensional or constructivist theories of emotion that share our view that the different emotions as they are understood in common-sense psychology are unlikely to map onto distinct neural circuits. Constructionists argue instead for an account of the different emotions as constructed out of the activity and interaction among domain-general neural networks. However we argued that constructionists may face a similar problem to the discrete emotion theorists they oppose. They may find that the domain-general networks to which they appeal likewise do not have fixed and permanent functions, but may shift their functions in ways that depend on context.

The moral we think of this considerations about brain regions and networks not having fixed and permanent functions is that we need to think of cognitive function in the brain as context-sensitive. We then turned our attention to offering an account of this context-sensitivity. We argued for a view of affect as states of action readiness involving the whole body of the organism. States of action readiness manifest in the body as forms of arousal that are either positively or negatively valued. These bodily states prepare the organism to respond to relevant opportunities and challenges in the environment. These states of action readiness are tracked by interoceptive processes in the brain. The salience network very likely plays a central role in this process (Menon and Uddin, 2010). We then argued that patterns of large scale activity take shape in the brain in ways that are driven by the states of action readiness in the body as a whole. These states of action readiness are elicited by relevant affordances in the environment, and make the organism ready to respond to relevant affordances.

So far our arguments have focused entirely on emotion and how best to understand emotion in the brain. We take our argument however to point to the more general conclusion that cognitive function is best investigated at the level of the whole brain-body-environment system. We take such a conclusion to follow from what we’ve already argued about emotion and cognition interactions in the brain. We’ve seen above that there any separation of emotional and cognitive processes in the brain doesn’t hold up in reality. The brain areas that neuroimaging studies identify as being active when people perform tasks that engage emotional and cognitive processes turn out to be in constant and continuous interaction. We’ve also argued that emotional processes take place in the living body of the organism in its interactions with an environment rich with affordances. Given that there is no separating emotion and cognition it follows that cognitive functions likewise deeply depend on the whole living body of the organism in its engagement with an environment rich with affordances.

## Conflict of Interest Statement

The authors declare that the research was conducted in the absence of any commercial or financial relationships that could be construed as a potential conflict of interest.
